# Evolutionary paths to mammalian longevity through the lens of gene expression

**DOI:** 10.15252/embj.2023114879

**Published:** 2023-07-31

**Authors:** Ulaş Işıldak, Handan Melike Dönertaş

**Affiliations:** ^1^ Leibniz Institute on Aging ‐ Fritz Lipmann Institute (FLI) Leibniz Germany

**Keywords:** Biomarkers, Chromatin, Transcription & Genomics, Evolution & Ecology

## Abstract

The natural variation in mammalian longevity and its underlying mechanisms remain an active area of aging research. In the latest issue of *The EMBO Journal*, Liu *et al* (2023) analyze gene expression levels in 103 mammalian species across three tissues, revealing tissue‐specific associations between gene expression patterns and longevity. Remarkably, the study suggests that methionine restriction, a strategy shown to increase lifespan, may extend beyond artificial interventions and is similarly employed by natural selection.

## Graphical abstract

A new large‐scale transcriptomic study of 103 species reveals methionine restriction and other pathways are associated with increased lifespan in mammals.

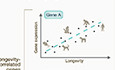

Mammals exhibit a wide range of maximum lifespans, from 2 to 3 years in certain shrew species to remarkable longevity in species like bowhead whales, which can live for over 200 years. Investigating the molecular foundations of this diversity yields valuable insights into aging and age‐related functional decline. Comparative genomic studies have primarily focused on specific taxa and particularly long‐lived species, such as naked‐mole rats (Kim *et al*, 2011), whales (Keane *et al*, 2015), and bats (Seim *et al*, 2013). These studies have highlighted the critical roles of DNA repair, cell cycle regulation, mitochondrial function, and tumor suppression. While common pathways have emerged, each study has underscored distinct factors that govern lifespan regulation across diverse taxa. Notably, Cui *et al* (2019) took an orthogonal approach and focused on a short‐lived species, turquoise killifish, and closely related species with varying lifespans. They proposed the relaxation of purifying selection as a general mechanism for the evolution of shorter lifespans.

Another avenue for regulating longevity is through gene expression rather than genetic variation. The transcriptome, which offers a more direct link to phenotypic evolution and can undergo rapid evolutionary changes compared to genomics, holds promise for unraveling new insights into lifespan regulation, potentially with tissue‐specific implications. Comparative transcriptomics studies, which have also primarily focused on long‐lived species, specific taxa, or a limited number of species, have identified key pathways, including energy metabolism, DNA damage repair, autophagy, immunity, transcriptional regulation, proteostasis, and tumor suppression (Seim *et al*, 2014; Fushan *et al*, 2015; Huang *et al*, 2019). Despite their smaller number of species characterized, these studies demonstrate the utility of comparative approaches in understanding the role of the transcriptome in lifespan regulation while revealing diverse mechanisms at play. Therefore, conducting a more comprehensive study encompassing a broader range of mammalian taxa becomes crucial to comprehend the overarching mechanisms governing lifespan and identify those consistently favored by natural selection.

In a new research paper published in *The EMBO Journal*, Liu *et al* (2023) conducted a comparative transcriptomics analysis across the mammalian tree of life to unveil the regulation of lifespan at the gene expression level. To this end, the authors integrated a very comprehensive gene expression dataset, covering liver, kidney, and brain tissues of 103 mammalian species, 56 of which were sequenced in this study. They first identified genes whose expression levels significantly correlate with longevity traits (Fig 1, middle panel). Among these genes, *PNMA1* was consistently positively correlated with longevity in all three tissues. While previous studies associated *PNMA1* with apoptosis (Jiang *et al*, 2014), its role in the context of aging remains unclear. The results presented by Liu and colleagues indicate its potential involvement in the evolution of lifespan and suggest it as a promising target for anti‐aging interventions. Longevity‐correlated genes were also enriched for transcription and translation fidelity pathways in all three tissues, suggesting their importance in ensuring mammalian longevity. Surprisingly, only a small portion of longevity‐correlated genes overlapped with the GenAge genes, which modulate lifespan or aging phenotypes in laboratory animals. This limited overlap implies that most of these aging‐related genes may not serve as a basis for the evolution of longevity, while also highlighting a potential to follow the footsteps of evolution and explore longevity‐correlated genes for artificial interventions.

**Figure 1:**
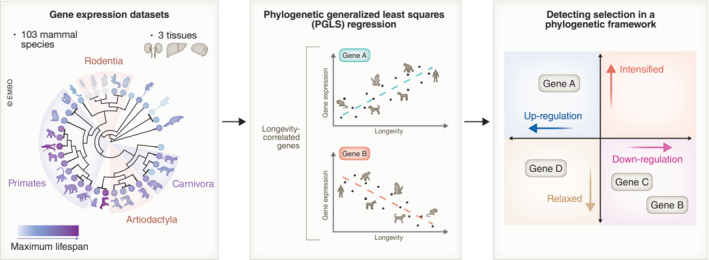
Graphical overview of the workflow adopted by Liu *et al* (2023) Integration of a comprehensive dataset from liver, kidney, and brain tissues of 103 mammal species with maximum lifespans ranging from about 3 years to over 200 years (left panel). Phylogenetic generalized least squares regression was conducted to explore the role of gene expression variation in longevity regulation, identifying genes whose expression levels significantly correlated with longevity traits as longevity‐correlated genes (middle panel). Selection intensities of longevity‐correlated genes were analyzed within a phylogenetic framework (right panel).

Next, Liu and colleagues conducted an integrated analysis of gene expression and selection in the genome to uncover the selective forces influencing the identified longevity‐associated genes. They found that the majority of longevity‐correlated genes were evolving under intensified or relaxed positive selection rather than purifying (i.e., negative) selection. This demonstrates that positive selection, which promotes the spread of beneficial alleles, might be the primary factor shaping the genes whose expression is correlated with longevity traits. Moreover, they found that species with longer lifespans tend to have more genes under intensified selection. In fact, about 62% of genes were identified as being subject to intensified selection in long‐lived mammals. Longevity‐correlated genes that are under intensified selection showed enrichment for the methionine salvage pathway. This pathway is known to be involved in the regeneration of methionine, an essential amino acid, and the production of polyamines like spermidine. Previous studies have shown that methionine restriction reverses inflammation, reduces DNA damage, and is associated with extended lifespan (Bárcena *et al*, 2018). These findings suggest that genes associated with methionine restriction play an important role in the evolution of lifespan extension. This highlights a shared strategy between natural selection and human‐induced laboratory interventions, such as drug treatments or genetic manipulations, aimed at anti‐aging and the regulation of lifespan.

Liu *et al* (2023) present a combination of extensive data collection and an innovative approach to comparative genomics of aging by merging genomics and transcriptomics. Through the integration of diverse data from multiple species, they have laid a strong foundation for future studies. The authors have identified numerous new candidate genes that may play crucial roles in the evolution of lifespan, thereby providing potential targets for anti‐aging interventions. Additionally, their work highlights the organ‐specific nature of the evolutionary correlation between gene expression and longevity, which is characterized by polygenic selection. While shedding light on the intricate interplay among natural selection, gene expression, and lifespan regulation, this newfound understanding also prompts further areas of inquiry. One intriguing area for future investigation lies in uncovering the evolutionary patterns of sex differences in gene expression and longevity. Additionally, examining a wider variety of tissues is crucial to determine which ones play a pivotal role in lifespan regulation and identify biological processes related to lifespan that are shared or specific to certain tissues. It is worth noting that numerous genes known to regulate lifespan in laboratory settings are conserved and only a small subset of them is associated with longevity across species. The next step is to integrate these two domains—the natural and artificial variations in lifespan—to identify more practical targets for promoting healthy aging and extending lifespan. Furthermore, future research should incorporate age‐series transcriptomics data and explore the intricate relationship between longevity and age‐related patterns of gene expression. Building upon the groundwork laid by *Liu et al*, the path ahead involves deepening our knowledge of lifespan regulation rather than solely accumulating more data. Through these efforts, we can gain a more comprehensive understanding of the complex interplay among gene expression, natural selection, and the regulation of lifespan in diverse mammalian species.

## References

[embj2023114879-bib-0001] Bárcena C , Quirós PM , Durand S , Mayoral P , Rodríguez F , Caravia XM , Mariño G , Garabaya C , Fernández‐García MT , Kroemer G *et al* (2018) Methionine restriction extends lifespan in progeroid mice and alters lipid and bile acid metabolism. Cell Rep 24: 2392–2403 30157432 10.1016/j.celrep.2018.07.089 PMC6130051

[embj2023114879-bib-0002] Cui R , Medeiros T , Willemsen D , Iasi LNM , Collier GE , Graef M , Reichard M , Valenzano DR (2019) Relaxed selection limits lifespan by increasing mutation load. Cell 178: 385–399 31257025 10.1016/j.cell.2019.06.004

[embj2023114879-bib-0003] Fushan AA , Turanov AA , Lee S‐G , Kim EB , Lobanov AV , Yim SH , Buffenstein R , Lee S‐R , Chang K‐T , Rhee H *et al* (2015) Gene expression defines natural changes in mammalian lifespan. Aging Cell 14: 352–365 25677554 10.1111/acel.12283 PMC4406664

[embj2023114879-bib-0004] Huang Z , Whelan CV , Foley NM , Jebb D , Touzalin F , Petit EJ , Puechmaille SJ , Teeling EC (2019) Longitudinal comparative transcriptomics reveals unique mechanisms underlying extended healthspan in bats. Nat Ecol Evol 3: 1110–1120 31182815 10.1038/s41559-019-0913-3

[embj2023114879-bib-0005] Jiang S‐H , He P , Ma M‐Z , Wang Y , Li R , Fang F , Fu Y , Tian G‐A , Qin W‐X , Zhang Z‐G (2014) PNMA1 promotes cell growth in human pancreatic ductal adenocarcinoma. Int J Clin Exp Pathol 7: 3827–3835 25120759 PMC4128994

[embj2023114879-bib-0006] Keane M , Semeiks J , Webb AE , Li YI , Quesada V , Craig T , Madsen LB , van Dam S , Brawand D , Marques PI *et al* (2015) Insights into the evolution of longevity from the bowhead whale genome. Cell Rep 10: 112–122 25565328 10.1016/j.celrep.2014.12.008 PMC4536333

[embj2023114879-bib-0007] Kim EB , Fang X , Fushan AA , Huang Z , Lobanov AV , Han L , Marino SM , Sun X , Turanov AA , Yang P *et al* (2011) Genome sequencing reveals insights into physiology and longevity of the naked mole rat. Nature 479: 223–227 21993625 10.1038/nature10533 PMC3319411

[embj2023114879-bib-0008] Liu W , Zhu P , Li M , Li Z , Yu Y , Liu G , Du J , Wang X , Yang J , Tian R *et al* (2023) Transcriptomic and selection signatures of longevity in mammals. EMBO J 42: e112740 10.15252/embj.2022112740 PMC10476176 37427458

[embj2023114879-bib-0009] Seim I , Fang X , Xiong Z , Lobanov AV , Huang Z , Ma S , Feng Y , Turanov AA , Zhu Y , Lenz TL *et al* (2013) Genome analysis reveals insights into physiology and longevity of the Brandt's bat *Myotis brandtii* . Nat Commun 4: 2212 23962925 10.1038/ncomms3212 PMC3753542

[embj2023114879-bib-0010] Seim I , Ma S , Zhou X , Gerashchenko MV , Lee S‐G , Suydam R , George JC , Bickham JW , Gladyshev VN (2014) The transcriptome of the bowhead whale *Balaena mysticetus* reveals adaptations of the longest‐lived mammal. Aging 6: 879–899 25411232 10.18632/aging.100699 PMC4247388

